# Optimizing mating strategies to maximize genetic diversity in the mhorr gazelle (*Nanger dama mhorr*) ex situ breeding program

**DOI:** 10.1186/s40850-026-00264-4

**Published:** 2026-04-27

**Authors:** Sonia Domínguez, Juan Pablo Gutiérrez, Eulalia Moreno, Isabel Cervantes

**Affiliations:** 1 Estación Experimental de Zonas Áridas-CSIC, Ctra. De Sacramento s/n, La Cañada de San Urbano, Almería, 04120 Spain; 2 Department of Animal Production, Faculty of Veterinary, UCM, Avda. Puerta de Hierro s/n, Madrid, 28040 Spain

**Keywords:** Critically endangered, Captive breeding, Dama gazelle, Mating strategies, Genetic diversity, Effective population size

## Abstract

To minimize coancestry among the offspring by equalising parents’ contributions is currently one of the most widely used methods to maximize the genetic diversity in conservation programs of threatened species. The objective of this study was to compare the performance of 22 different mating strategies to limit the loss of genetic diversity in a real captive population of mhorr gazelle (*Nanger dama mhorr*) via computer simulations. This objective was achieved by monitoring the evolution of the effective population size (*Ne*) throughout 15 generations. The studied population consisted of 87 breeding animals (25 males and 62 females), from a total pedigree of 3059 records. The scenarios were designed according to different assumptions: the use of parents coancestry or offspring coancestry, the use of the coefficients themselves in calculations or their increases, and the number of males and females involved in the mating. The results for this captive population showed that strategies to minimize the parent’s coancestry were the best in the short term, but mixed strategies to minimize coancestry of both parents and offspring were better in the medium and long term when a weight to coancestry in the parents’ generation between 5% and 50% was applied. Furthermore, it was observed that these mixed strategies improved their performance when all females in the population participated in breeding, but not all males. These results suggest that each managed population may need different mating strategies, considering its generation interval, the species’ breeding system, the expected time horizon of the conservation program, or other species-specific considerations of the population.

**Supplementary Information:**

The online version contains supplementary material available at 10.1186/s40850-026-00264-4.

## Introduction

Threatened species suffer a greater loss of genetic diversity due to their small size population and increased relatedness between individuals [7]. Ex situ breeding programs aim to maintain self-sustaining populations that prevent the complete extinction of the species and release new individuals into the wild, when suitable natural habitat exists and the threat to the species in the wild is controlled [56]. From a genetic point of view, ex situ breeding programs should retain maximum genetic variability of the population, as it will determine its future adaptive potential (Falconer 1981) and limit the accumulation of inbreeding, which can lead to a reduction in fitness [36]. To this end, careful choice of breeding animals and effective mating strategies are essential to maintain genetic integrity of target populations, as well as monitoring the genetic consequences of these captive breeding programs and the genetic diversity obtained in the offspring [55]. The effective population size (*Ne*) is a widely used parameter to monitor the status of genetic diversity in breeding populations [34], as it provides information on the rate of inbreeding [50] and genetic changes due to genetic drift [35]. The effective population size of a population refers to the hypothetical number of breeding animals in an idealized population that would lead to the actual increase in inbreeding if they contribute equally to the next generation [58]. Compared to other parameters that assess genetic diversity, whether based on founder contributions (e.g. founder genome equivalent), or the variety of alleles and genes in the population (e.g. gene diversity), the inverse relationship with the accumulation of inbreeding offered by the effective population size provides valuable additional information about the potential deleterious effects on the population [40, 49]. In addition to the increase of inbreeding, the effective population size also measures the degree of erosion in genetic variability caused by genetic drift, which depends greatly on population size [28]. Thus, in small populations, like many of those kept in captive breeding programs, random changes in allele frequencies caused by genetic drift are stronger and faster, resulting in a lower effective population size; while in large populations genetic drift has less impact over generations and results in higher effective population sizes [1, 22].

The dama gazelle (*Nanger dama*) is a critically endangered antelope native to North Africa [29]. Indiscriminate hunting and habitat destruction have been the main causes of its decline, leading even to the extinction in the wild of the western subspecies, the mhorr gazelle (*Nanger dama mhorr*) [16]. The complete disappearance of the mhorr gazelle was avoided with the creation in 1971 of an ex situ breeding program from some of the last living specimens at “La Hoya” Experimental Field Station (EEZA-CSIC), in Almeria (Spain). The captive population has gradually increased to over 300 individuals at the present, distributed across different centres in Europe, North America and the Arabian Peninsula [13]. The ultimate goal of this ex situ breeding program is the restoration of the mhorr gazelle in its natural habitat. The dama gazelle is a social species usually forming herds made up of one adult male, several females and their youngsters. It follows a polygynous mating system, where dominant males becoming territorial during the mating season [6]. Females reach sexual maturity at 9–12 months of age, and males between 18 and 24 months. The gestation has a duration of 6.5 months and only one calf is born per birth [4].

In the present study, the performance of different mating strategies to limit the losses of genetic diversity was compared in a real captive population of mhorr gazelle via computer simulations. The effect of different aspects on the effectiveness of the mating schemes was evaluated, such as the use of parents coancestry or offspring coancestry, the number of males and females involved in the mating, and the use of the coefficients themselves in calculations or their increases. In all cases, the effective population size and the inbreeding coefficient were used as measures of the evolution of genetic diversity in the population, both in the short and long term. Although the effective population size selected for monitoring is based on individual increase in inbreeding, we also considered it important to measure the average individual inbreeding coefficient given its high value (0.28) in this study population [14] and the inbreeding depression effects already observed [15].

## Materials and methods

### Study population

The mating group used for this work included all living animals present at the Almeria breeding centre at the time of the study (December 31, 2024), to recreate a situation as realistic as possible. This reference population consisted of 87 animals (25 males and 62 females) born between 2010 and 2024 (see details in Table 1), from a total pedigree of 3059 records. The population of Almeria was chosen because it is the largest in the world and maintains the reproduction of several breeding groups simultaneously, while other zoological institutions only hold one breeding group, of usually no more than 5–10 animals. In Almeria, breeding herds are generally formed with 1 male and 4–8 females. Breeding males are selected following a mating strategy of minimum coancestry of offspring and they are changed annually in each group of females to maximize genetic diversity.

**Table 1:** Age structure of the reference population of Almeria according to the year of birth

Year of birth	Males	Females	Total animals
2010	0	2	2
2011	0	1	1
2012	0	2	2
2013	0	4	4
2015	1	3	4
2016	1	8	9
2018	0	6	6
2019	1	12	13
2020	1	3	4
2021	3	1	4
2022	3	4	7
2023	5	3	8
2024	10	13	23
Total reference population	25	62	87

The mhorr gazelle living population of Almeria has an average generation interval [32] of 5.87 years, and a number of equivalent complete generations [41] of 8.72, which indicates a high pedigree completeness level. In this living population the average value of individual inbreeding coefficient [42] is 0.28. The effective population size based on individual increase in inbreeding ($\:\stackrel{-}{{N}_{ei}}\:$) [27] and based on individual increase in coancestry ($\:\stackrel{-}{{N}_{ec}}\:$) [10] are, respectively, 13.2 and 12.1. The ratio $\:\stackrel{-}{{N}_{ec}}/\stackrel{-}{{N}_{ei}}$ close to one evidences that Almeria does not have a structured population [14].

### Mating strategies

Different mating strategies were tested by minimizing a function *Fx* [46]. The strategies were classified into three groups according to their purpose:

1. Strategies to minimize the parent’s coancestry (or inbreeding):
  Strategy of minimum parent’s coancestry (F): each animal mates with the least related individual of the opposite sex, so that inbreeding of the offspring is minimal. In this strategy $\:Fx=\:\sum\:{C}_{jk}$, where $\:{C}_{jk}$ is the coancestry between male *j* and female *k*. Two alternatives were tested:
  Strategy F0: all females contribute offspring to the next generation, but not all males.
  Strategy F1: all females and all males contribute offspring to the next generation.
  Strategy of minimum increase in parent’s coancestry (ΔF): since coancestry accumulates per generation, under this strategy differences in pedigree depth of individuals were considered. Thus, $\:Fx=\:\sum\:{\varDelta\:C}_{jk}$, where $\:{\varDelta\:C}_{jk}=1-\:\sqrt[\frac{{g}_{j}+{g}_{k}}{2}]{1-\:{C}_{jk}}$ [30], where g_j_ and g_k_ are the equivalent discrete generations known for individuals *j* and *k*, and *C*_*jk*_ is the inbreeding of a descendent of these two individuals [41]. Again, two alternatives were tested:
  Strategy ΔF0: all females contribute offspring to the next generation, but not all males.
  Strategy ΔF1: all females and all males contribute offspring to the next generation.
  Strategy of minimum parent’s coancestry weighted by contributions (Fw): this strategy takes into account the current representation of each individual in the mating group and penalizes those who are already highly represented. In this case, $\:Fx=\:\sum\:{{m}_{j}C}_{jk}{m}_{k}$, where *m*_*j*_ (and *m*_*k*_) is the mean coancestry between the individual *j* (and *k*) and all the other animals in the mating group. Two alternatives were checked:
  Strategy Fw0: all females contribute offspring to the next generation, but not all males.
  Strategy Fw1: all females and all males contribute offspring to the next generation.
  Strategy of minimum increase in parent’s coancestry weighted by contributions (ΔFw): this strategy simultaneously accounts possible differences in pedigree depth and representation within the mating group. Function $\:Fx=\:\sum\:{{\varDelta\:m}_{j}\varDelta\:C}_{jk}\varDelta\:{m}_{k}$, where Δ*m*_*j*_ (and Δ*m*_*k*_) is the mean of the increase in coancestry between the individual *j* (and *k*) and all the other animals involved in the mating plan. Likewise, two alternatives were studied:
  Strategy ΔFw0: all females contribute offspring to the next generation, but not all males.
  Strategy ΔFw1: all females and all males contribute offspring to the next generation.

2. Strategies to minimize the offspring’s coancestry:
  Strategy of minimum offspring’s coancestry (C): unlike previous methods that minimize the inbreeding of the descendants, this strategy seeks that the average kinship between all the resulting descendants is minimal. Here $\:Fx=\:\sum\:{C}_{lm}$, where $\:{C}_{lm}$ is the coancestry between two descendants *l* and *m* of the mating design. In this case, three alternatives were analyzed:
  Strategy C0: all females contribute offspring to the next generation, but not all males.
  Strategy C1: all females and all males contribute offspring to the next generation.
  Strategy C2: not all females and not all males participated with offspring for the next generation, while others had a higher number of descendants.
  Strategy of minimum increase in offspring’s coancestry (ΔC): pedigree depth information is taken into account to calculate kinship. In this strategy $\:Fx=\:\sum\:{\varDelta\:C}_{lm}$, where $\:{\varDelta\:C}_{lm}$ is the increase in the coancestry between two descendants l and *m* of the mating design. Three alternatives were tested:
  Strategy ΔC0: all females contribute offspring to the next generation, but not all males.
  Strategy ΔC1: all females and all males contribute offspring to the next generation.
  Strategy ΔC2: not all females and not all males participated with offspring for the next generation.

3. Strategies to minimize a combination of the parent’s coancestry and the offspring’s coancestry:
  Mixed strategy (M): combines information from two generations, parents and offspring. Thus, function $\:Fx=\:p\sum\:{C}_{jk}+(1-\:{p}_{1})\sum\:{C}_{lm}$ was minimized, where again $\:{C}_{jk}$ is the coancestry between male *j* and female *k*, and therefore the inbreeding of their offspring, and $\:{C}_{lm}$ is the coancestry between two descendants *l* and *m* of the mating design. In this equation *p* is a value between 0 and 1 indicating the weight that will be given to coancestry in the parents’ generation. The following values were tested for *p*: 0.01 (M 1–99), 0.05 (M 5–95), 0.50 (M 50–50), and 0.95 (M 95 − 5). Thus, the first value indicated in the strategy refers to the weight given to minimize parent’s coancestry, and the second value, to the weight to minimize the offspring’s coancestry. Two different alternatives were studied:
  Strategy M0: all females contribute offspring to the next generation, but not all males.
  Strategy M2: not all females and not all males participated with offspring for the next generation.

Different alternatives were tested regarding the number of males and females involved in the mating. The benefits of having the largest possible breeding stock to maximize genetic diversity are clear, because it increases the possibility of maintaining a greater variety of alleles, reduces the impact of genetic drift and minimizes inbreeding. However, this study also compared other strategies where not all males or all females necessarily contribute as breeders to the next generation, since there may be populations with overrepresented individuals where increasing their number of descendants is not positive for the general genetic diversity. On the contrary, leaving out some breeding animals could be optimal from the point of view of the function to be minimized, but the participation of some alleles that could improve the result in the long term could be lost.

### Computations performed

A total of 22 mating strategies were tested. The optimal mating design was found using a simulated annealing algorithm [59]. Fifty replicates of each strategy were conducted during 15 discrete generations. According to a previous study of Ojeda-Marín et al., [46] in a species with a similar generation interval and taxonomically close to the mhorr gazelle, this number of discrete generations was sufficient to compare the proposed strategies, since no differences were observed between the results in the fifteenth generation and the twentieth generation, while the simulation process is extremely time consuming. Each generation was formed by a random number of animals simulated from a Poisson distribution of the reference population to be mated, where the number of males was assumed to be equal to or lower than the number of females, and a similar sex ratio was maintained throughout the generations by randomly being born as male or female according to their current representation in the population.

The performance of the mating strategies was evaluated by the evolution of the effective population size over the 15 generations. The value of the effective population size used to monitor the strategies was the one based on individual increase in inbreeding $\:\left(\stackrel{-}{{N}_{ei}}=\:\:\:\frac{1}{2\stackrel{-}{{\Delta\:}F}}\right)$ [26, 27] instead of the effective population size based on individual increase in coancestry $\:\left(\stackrel{-}{{N}_{ec}}=\:\:\:\frac{1}{2\stackrel{-}{{\Delta\:}C}}\right)$ [10], because problems of inbreeding depression were recently described in this ex situ population [15] and $\:\stackrel{-}{{N}_{ei}}$ minimizes the rate of inbreeding (ΔF) due to its inverse relationship [8]. In addition, high $\:\stackrel{-}{{N}_{ec}}$ values ​​could mask difficulties in the viability of populations with low relatedness between individuals, but with high inbreeding [45].

All the analyses were computed using the open access ENDOG program v4.9 [25]. Differences between strategies were considered significant when the standard error of the effective population size multiplied by 1.96 defined an interval that did not include 0.

## Results

**Fig. 1:**
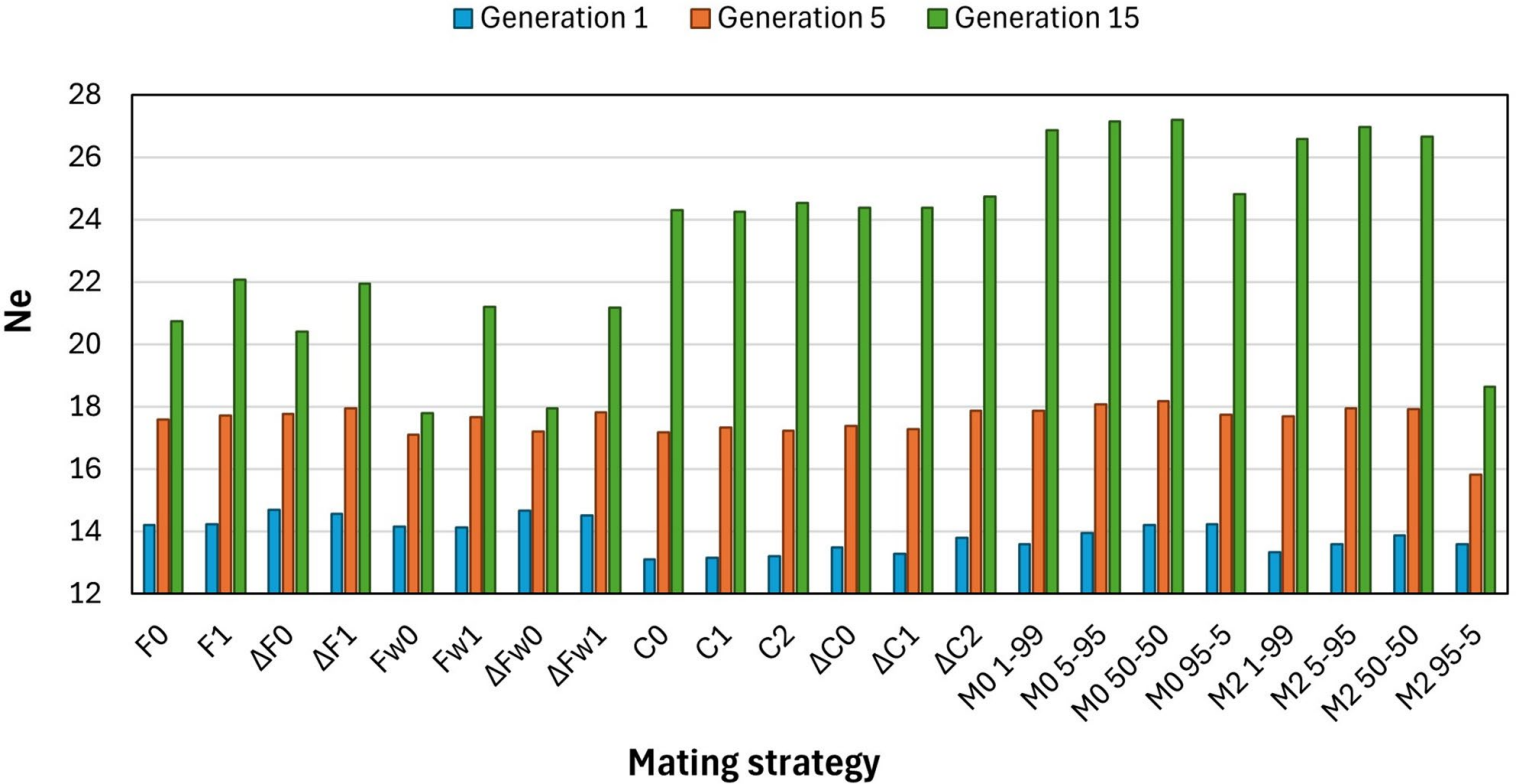
Effective population size obtained by the different strategies in generations 1, 5, and 15: based on minimum parent’s coancestry (F0 and F1), on minimum increase in parent’s coancestry (ΔF0 and ΔF1), on weighted parent’s coancestry (Fw0 and Fw1), on weighted increase in parent’s coancestry (ΔFw0 and ΔFw1), on minimum offspring’s coancestry (C0, C1 and C2), on minimum increase in offspring’s coancestry (ΔC0, ΔC1 and ΔC2), and on mixed information from parent’s coancestry and offspring’s coancestry (M0 1–99, M0 5–95, M0 50–50, M0 95 − 5, M2 1–99, M2 5–95, M2 50–50 and M2 95 − 5)

Figure 1 shows the effective population size in generations 1, 5 and 15 of the different simulated strategies. In the short term (generation 1), the best results were from strategies ΔF0 and ΔFw0. In contrast, the worst strategies were C0, C1, C2, ΔC1 and M2 1–99. In the medium term (generation 5), the differences between the Ne values ​​were quite small, although the strategy M0 50–50 was significantly the best, followed by strategy M0 5–95. The worst performance was obtain using strategy M2 95 − 5. In the long term (generation 15), strategies M0 5–95 and M0 50–50 showed the best effective population size values. The strategies that gave higher losses of genetic variability in the long term were Fw0, ΔFw0 and M2 95 − 5.

**Fig. 2:**
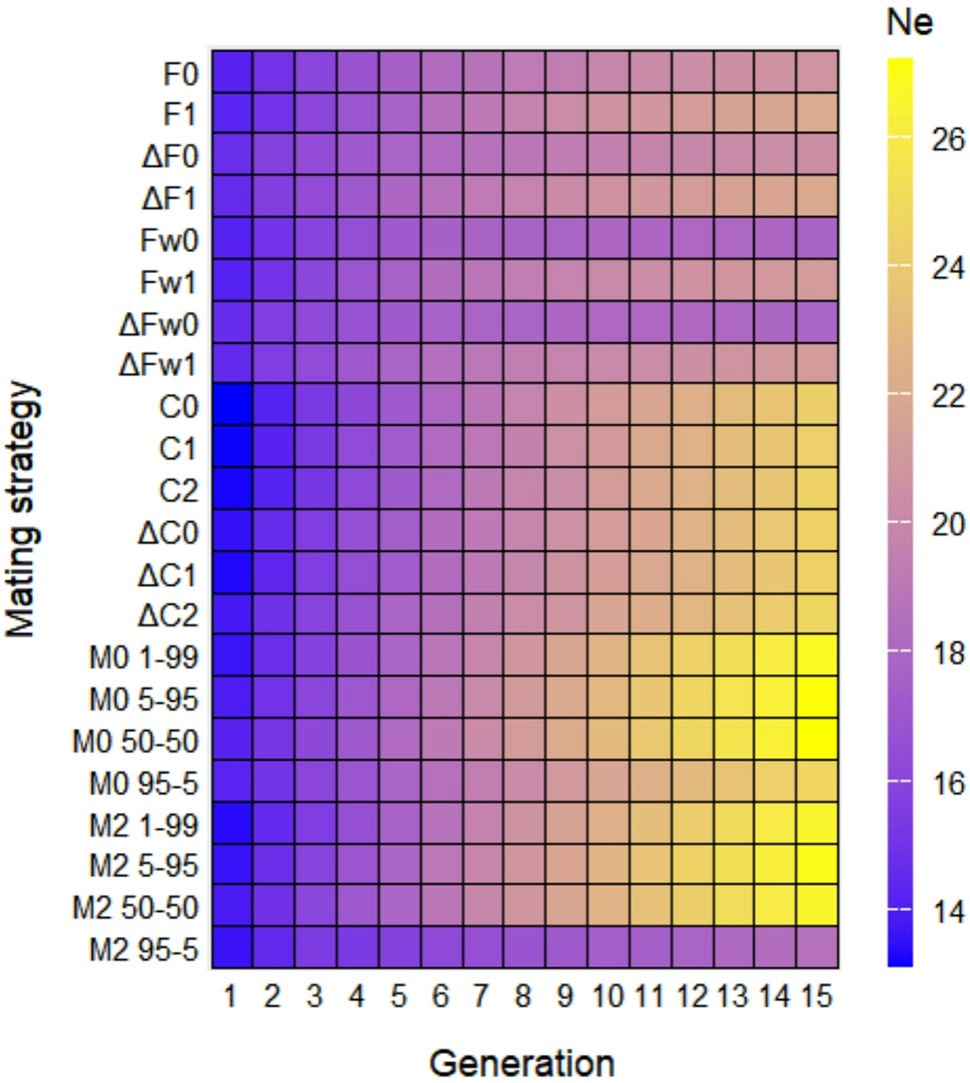
Evolution of effective population size obtained by the different strategies throughout 15 generations: based on minimum parent’s coancestry (F0 and F1), on minimum increase in parent’s coancestry (ΔF0 and ΔF1), on weighted parent’s coancestry (Fw0 and Fw1), on weighted increase in parent’s coancestry (ΔFw0 and ΔFw1), on minimum offspring’s coancestry (C0, C1 and C2), on minimum increase in offspring’s coancestry (ΔC0, ΔC1 and ΔC2), and on mixed information from parent’s coancestry and offspring’s coancestry (M0 1–99, M0 5–95, M0 50–50, M0 95 − 5, M2 1–99, M2 5–95, M2 50–50 and M2 95 − 5)

The evolution of effective population size throughout generations according to the mating strategies studied is shown in Fig. 2. Overall, mixed strategies had the best performance in the long term, followed by the strategies that only minimize the offspring’s coancestry, and the strategies that minimize the parent’s coancestry obtain the lower effective population size values.

Comparing the number of males and females involved in the mating, differences were also observed between the strategies in generation 15. Methods that minimize the parent’s coancestry improved when all females and all males participated in breeding (F1, ΔF1, Fw1 and ΔFw1), compared to those where all females contribute offspring to the next generation, but not all males (F0, ΔF0, Fw0 and ΔFw0). Among the strategies that minimize the offspring’s coancestry, the effective population size values ​​were slightly better when not all females and not all males contribute offspring to the next generation (C2 and ΔC2), but these differences were only significant for strategy ΔC2. In the case of the mixed strategies, the results were always better when all females were expected to contribute to the next generation, but not all males (M0), than when not all females nor all males participate in breeding (M2).

Regarding the strategies that minimize the parent’s coancestry, methods that minimized increases (ΔF0, ΔF1, ΔFw0 and ΔFw1) instead of the coefficients themselves (F0, F1, Fw0 and Fw1) had better results in the short term, but no significant differences were obtain in the long term. Within the methods that minimize the offspring’s coancestry, strategies ΔC0 and ΔC2 achieved significantly higher effective population size than strategies C0 and C2 in the short term. Similarly, in no case significant differences were found in the long term.

**Fig. 3:**
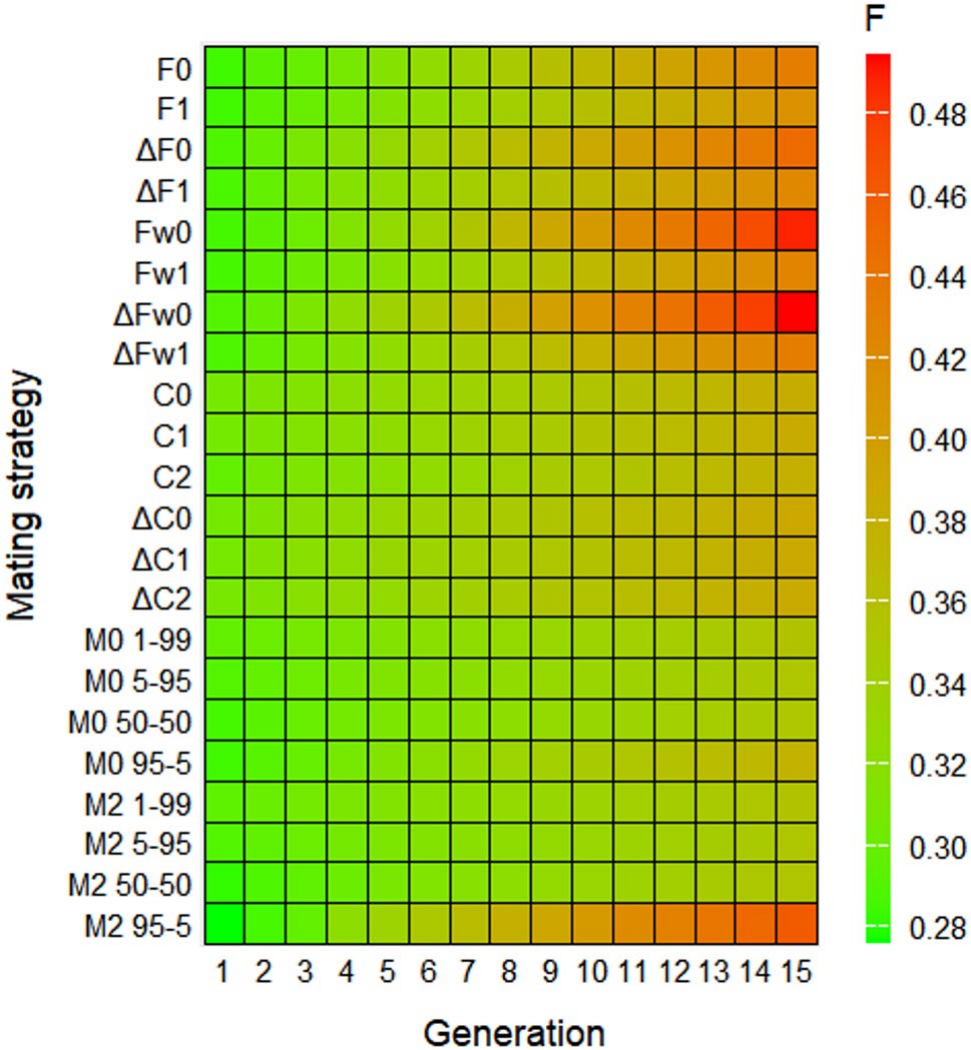
Evolution of inbreeding coefficient obtained by the different strategies throughout 15 generations: based on minimum parent’s coancestry (F0 and F1), on minimum increase in parent’s coancestry (ΔF0 and ΔF1), on weighted parent’s coancestry (Fw0 and Fw1), on weighted increase in parent’s coancestry (ΔFw0 and ΔFw1), on minimum offspring’s coancestry (C0, C1 and C2), on minimum increase in offspring’s coancestry (ΔC0, ΔC1 and ΔC2), and on mixed information from parent’s coancestry and offspring’s coancestry (M0 1–99, M0 5–95, M0 50–50, M0 95 − 5, M2 1–99, M2 5–95, M2 50–50 and M2 95 − 5)

Finally, Fig. 3 shows the evolution of inbreeding coefficient by generation across methodologies. It can be noticed that there is a direct inverse relationship between the strategies that have the highest values ​​of effective population size and the lowest inbreeding coefficients as expected.

All the numeric results of the effective population size and the inbreeding coefficient, along with their standard errors, obtained from each strategy throughout the generations are compiled in Table S1 and Table S2, respectively. The confidence intervals of the statistical analyses are also included In Table S3.

## Discussion

It is widely recognized that the optimal method for maximizing the genetic diversity in the conservation programs managed using pedigree information is to minimize coancestry among the offspring by making the contributions of parents more equal [20, 30, 53]. However, it has been suggested that the best mating strategy can vary depending on the managed population and each case needs to be studied individually [46]. With this approach, the present work has intended to analyze how the genetic diversity of a real captive population of a critically endangered species varies according to the different mating strategies raised.

Firstly, although the strategies that minimize the parent’s coancestry were the best in the short term, their results were the worst in the long term, apart from those of the M2 95 − 5 strategy. These strategies that focus, exclusively or to a high percentage, on minimizing the inbreeding of offspring, mate each animal with the least related individual of the opposite sex and obtain a high value of the effective population size in the first generation. However, as consequence, the second generation is made up of individuals that, although they have a low average inbreeding, are in a large proportion siblings or half-siblings, which is to say there is a high average kinship between all of them. This initial and significant loss of genetic diversity then becomes unrecoverable in subsequent generations, preventing the effective population size from reaching better values ​​as with other strategies. It is also interesting to note how, despite the poor results of these strategies in the long-term, when all females and all males contribute to the next generation, the effective population size is clearly better than when all females contribute, but not all males. Therefore, a greater participation of breeding animals, both males and females, helps to reduce the levels of inbreeding and slow down the loss of genetic diversity [47, 57]. Methods that minimized increases instead of the coefficients themselves were beneficial in the short term, but not in the long term. In the first generations, the use of increase in parent’s coancestry allows standardizing the inbreeding coefficients based on the pedigree completeness [10]. Nevertheless, this is no longer an advantage in the latest generations, when the amount of genealogical information for all animals has become equal.

In second place, the strategies that minimize the offspring’s coancestry has the lowest effective population size values in the short term, besides the mixed strategies M0 1–99 and M2 1–99, that also focus preferably on minimizing this parameter. However, in the medium term these strategies were similar to those that minimize the parent’s coancestry and in the long term they surpassed them, which coincide with the most widely accepted theory at present [18]. In this case, not forcing the participation of either all males or all females improved the effective population size values, since preferential matings between relatives are favoured [52]. For these strategies, methods that minimize the increase in coancestry also showed an advantage in the first generations, but when the simulation of generations progresses, they are equated with the methods that minimize the coefficient of coancestry.

Finally, it can be seen how giving only minimal weight to coancestry in the parents’ generation of the mixed strategies (M0 1–99 and M2 1–99), the value of the effective population size increases significantly from the fifth generation onwards compared to the strategies that only minimize the offspring’s coancestry. Performance continues to improve further with a weight to coancestry in the parents’ generation of 5% and 50%, but then drops off at a weight of 95%. It should be noted that the simulations in this work did not include the potential negative impact on population viability of high inbreeding in early generations. Nevertheless, adding some weight to the parent’s coancestry, these mixed strategies avoid a high individual increase in inbreeding in animals at birth, and thus prevent the deleterious effects of inbreeding depression and a higher initial risk of population disappearance, while maximizing the effective population size in the long term. Regarding the number of individuals involved in the mating, it can be observed that forcing the participation of all females, but not all males, always had better results than not forcing the contribution of either sex to the next generation in these mixed strategies; arising an intermediate situation between the strategies that minimize the parent’s coancestry, where the contribution of all males and all females was the best option, and the strategies that minimize the offspring’s coancestry, where, on the contrary, the best performances were obtained when not all females nor all males participated with offspring for the next generation. In the wild, due to the territorial behaviour of males and the social organization of this species in harems, all females have the opportunity to contribute to the next generation, but only dominant and successful males in fights will be able to access the females and mate (van den Brink, 2018). Therefore, applying in captive matings this methodology that resembles the polygynous nature of the species seems reasonable, although in this case the selection criteria for breeding males are defined by the population managers and not by the physical superiority of the animals. Currently, most managers of captive breeding programs rely on genetic criteria that minimize coancestry of offspring and equalize the contributions of the parents. Nevertheless, human management, even without deliberate breeding goals, always favors certain traits in the animal population, leading to an almost inevitable unconscious selection [12, 54]. In addition, the simple and artificial environment of captivity prevents the natural behaviours of the species from being expressed and from being maintained by natural selection [11]. All of this can evoke in evolutionary changes such as adaptation to captivity or maladaptation to the wild [21, 23, 51]. Nevertheless, captive breeding programs can minimize the effects of unconscious selection on genetic diversity through careful genetic control of matings and by maximizing the effective population size [37], as well as propagating the population in large and natural enclosures that allow the greatest possible number of forms of natural selection [2]. Otherwise, it cannot be forgotten that in polygynous mating systems, where very few males obtain almost all of the matings, the high variance in male reproductive success may result in a low effective population size [43, 44]. Under extreme forms of polygyny, such as harem polygyny and dominance polygyny, this reduction can be even more marked. Therefore, the mhorr gazelle’s mating system makes this species naturally more prone to lower effective population sizes than other monogamous or random mating species.

Strategies based on parent coancestry would produce minimal coancestry in their offspring. However, ignoring the coancestry among the offspring, the genetic diversity of the population would pay so a heavy price, by discarding the participation of some breeding animals in the worst case, and keeping the unbalanced contribution of them in the best case, creating bottlenecks. Therefore, the optimal theoretical strategy in the long term falls on the mean global coancestry of the offspring, which is a strategy that trends to rebalance the contribution of the founders. However, there are several mating designs with identical representation of founders. Among them, those that have greater inbreeding in the offspring produce an equivalent higher loss in genetic diversity due to drift. Thus, those methods based on offspring coancestry that also place weight on parent coancestry become optimal.

The international community of zoos and wildlife breeding centers have pedigree analysis software available to carry out the demographic and genetic management of the populations they maintain [39]. Although traditionally most of these tools were designed for diploid species with sexual reproduction, they have subsequently incorporated new technological advances that better align with different species management (e.g. group living species) and breeding systems (e.g. hermaphroditic selfing, cloning or autozygous production of haploid offspring) [33, 38]. In the same way, a further step should be taken in the genetic management of wild species to adapt mating strategies to the particular needs of conservation programs, individually evaluating the best option for each population, to retain its maximum genetic diversity and minimize the potential deleterious effects of inbreeding. The optimal results obtained in this study with some of the mixed strategies reveal that there may be other approaches to mating design that are not currently being considered and that could be more efficient in avoiding the loss of genetic diversity. Therefore, it would be advisable for population managers to re-evaluate the mating schemes applied in their captive breeding programs, and include strategies that minimize coancestry of both parent and offspring as a new alternative. Using the same methodology as the present study, Ojeda-Marín et al., [46] showed that two populations with different levels of inbreeding did not always result in the same optimal mating strategies over generations. Conduct simulations on populations with different genetic structures would help to expand knowledge in this field and improve the genetic management of wild populations in captivity.

Another factor to take into account when choosing the optimal mating strategy is the expected time horizon. In this study population, given the species’ critical conservation status and the decades required to restore wild populations, maintaining captive populations will be essential to ensure the long-term viability of the conservation programme. Therefore, we compare a range of mating strategies projected over many generations. However, in populations of species with different degrees of threat, generation intervals or conservation objectives, comparing the performance of strategies in the short or medium term could be sufficient.

To conclude we can say that in this mhorr gazelle captive population the strategies M0 5–95 and M0 50–50 were the best overall in the medium and long term, and also had some of the highest effective population size values in the short term. Both strategies showed similar long-term performance, but M0 50–50 was significantly superior in the first and fifth generations than M0 5–95, so this would be the mating strategy of choice for this mhorr gazelle population. Although with some differences, these two strategies also had the best performance for the Cuvier’s gazelle population from the same ex situ breeding center [46], so applying a weight to coancestry in the parents’ generation between 5% and 50%, seems to have advantages in these particular cases. So far, the mating strategy applied in this mhorr gazelle population has been based exclusively on minimizing the offspring’s coancestry, but given the results obtained in this study, it would be highly recommended to replace it with the mixed strategy M0 50–50, whose performance has been shown to be clearly better than the method currently used by providing higher effective population size values. However, it should not be forgotten that coancestry coefficients can also be obtained from molecular information [9, 17] and used for monitoring genetic diversity in conservation programs [24]. Although molecular analyses have sometimes not differed from the results obtained from pedigree data [5, 31], or have even shown limited effectiveness if used alone [19], in other cases, they can be useful to contrast or complement the information obtained with genealogical data (Ito et al., 2017; [3]). Specifically, in polygynous species, contrary to expectations, molecular markers have shown that males contributed more genetic diversity than females, since they have a shorter reproductive lifetime and a higher turnover compared to females [48]. Therefore, it would be advisable to expand this work with molecular techniques to determine the definitive mating strategy for this population and the real levels of genetic diversity of each strategy across generations, as well as the effect of the contribution of different numbers of females and males on reproduction.

## Supplementary Information

Below is the link to the electronic supplementary material.

Supplementary Material 1

Supplementary Material 2

Supplementary Material 3

## Data Availability

The pedigree dataset analysed during the current study is available online: http://www.eeza.csic.es/documentos/Studbook_2021_Nanger_dama_mhorr.pdf.
