# Supplementary material for: Optimizing mating strategies to maximize genetic diversity in the mhorr gazelle (Nanger dama mhorr) ex situ breeding program
Source: BMC Zool. 2026 Apr 27;11:15. doi: 10.1186/s40850-026-00264-4 (PMC13154453; doi:10.1186/s40850-026-00264-4)
Supplement: Supplementary file 1 — Supplementary Material 1 [file 40850_2026_264_MOESM1_ESM.docx]

**SUPPLEMENTARY MATERIAL 1**

*Biodiversity and Conservation*

**Optimizing mating strategies to maximize genetic diversity in the mhorr gazelle (*Nanger dama mhorr*) ex situ breeding program**

Sonia Domínguez ^1^, Juan Pablo Gutiérrez ^2^, Eulalia Moreno ^1^ and Isabel Cervantes ^2^

^1^ Estación Experimental de Zonas Áridas-CSIC, Ctra. De Sacramento s/n, 04120 La Cañada de San Urbano, Almería, Spain

^2^ Department of Animal Production, Faculty of Veterinary, UCM, Avda. Puerta de Hierro s/n, 28040 Madrid, Spain

E-mail: [sdominguez@eeza.cisc.es](mailto:sdominguez@eeza.cisc.es)

**Table S1.** Evolution of effective population size and its standard error throughout 15 generations of each mating strategy in the reference population of Almeria: strategies that minimize the coancestry between the parents (a), strategies that minimize the coancestry of the offspring (b) and mixed strategies (c).

| **Generation** | **F0** | **F1** | **ΔF0** | **ΔF1** | **Fw0** | **Fw1** | **ΔFw0** | **ΔFw1** |
| --- | --- | --- | --- | --- | --- | --- | --- | --- |
| 1 | 14.22 ± 0.015 | 14.23 ± 0.014 | 14.70 ± 0.017 | 14.57 ± 0.014 | 14.15 ± 0.010 | 14.13 ± 0.009 | 14.66 ± 0.026 | 14.52 ± 0.014 |
| 2 | 14.99 ± 0.023 | 15.02 ± 0.025 | 15.73 ± 0.023 | 15.63 ± 0.019 | 15.02 ± 0.030 | 15.01 ± 0.021 | 15.56 ± 0.024 | 15.48 ± 0.016 |
| 3 | 15.99 ± 0.023 | 16.02 ± 0.027 | 16.39 ± 0.036 | 16.38 ± 0.020 | 15.95 ± 0.045 | 16.05 ± 0.023 | 16.24 ± 0.055 | 16.32 ± 0.021 |
| 4 | 16.84 ± 0.032 | 16.90 ± 0.030 | 17.14 ± 0.039 | 17.16 ± 0.022 | 16.50 ± 0.074 | 16.91 ± 0.025 | 16.71 ± 0.066 | 17.09 ± 0.033 |
| 5 | 17.58 ± 0.051 | 17.71 ± 0.047 | 17.76 ± 0.058 | 17.96 ± 0.040 | 17.11 ± 0.082 | 17.68 ± 0.044 | 17.20 ± 0.089 | 17.83 ± 0.046 |
| 6 | 18.29 ± 0.055 | 18.54 ± 0.051 | 18.27 ± 0.082 | 18.66 ± 0.059 | 17.58 ± 0.093 | 18.40 ± 0.066 | 17.63 ± 0.115 | 18.50 ± 0.059 |
| 7 | 18.79 ± 0.068 | 19.15 ± 0.065 | 18.67 ± 0.106 | 19.24 ± 0.074 | 17.80 ± 0.132 | 18.95 ± 0.089 | 17.78 ± 0.137 | 19.03 ± 0.069 |
| 8 | 19.20 ± 0.106 | 19.72 ± 0.068 | 18.94 ± 0.128 | 19.77 ± 0.081 | 17.80 ± 0.149 | 19.41 ± 0.103 | 17.84 ± 0.163 | 19.46 ± 0.085 |
| 9 | 19.47 ± 0.137 | 20.25 ± 0.077 | 19.33 ± 0.145 | 20.23 ± 0.090 | 17.86 ± 0.209 | 19.77 ± 0.103 | 17.97 ± 0.188 | 19.75 ± 0.114 |
| 10 | 19.88 ± 0.141 | 20.67 ± 0.081 | 19.62 ± 0.143 | 20.64 ± 0.096 | 17.97 ± 0.215 | 20.10 ± 0.106 | 18.02 ± 0.203 | 20.07 ± 0.116 |
| 11 | 20.18 ± 0.147 | 20.94 ± 0.119 | 19.80 ± 0.174 | 20.92 ± 0.103 | 17.97 ± 0.233 | 20.33 ± 0.126 | 18.04 ± 0.215 | 20.30 ± 0.129 |
| 12 | 20.40 ± 0.159 | 21.30 ± 0.124 | 19.96 ± 0.182 | 21.24 ± 0.110 | 18.02 ± 0.250 | 20.64 ± 0.144 | 18.13 ± 0.216 | 20.48 ± 0.143 |
| 13 | 20.53 ± 0.178 | 21.59 ± 0.138 | 20.14 ± 0.199 | 21.53 ± 0.118 | 18.02 ± 0.276 | 20.84 ± 0.160 | 18.05 ± 0.242 | 20.75 ± 0.135 |
| 14 | 20.66 ± 0.192 | 21.81 ± 0.150 | 20.38 ± 0.215 | 21.74 ± 0.117 | 17.96 ± 0.301 | 21.01 ± 0.165 | 18.00 ± 0.248 | 20.99 ± 0.147 |
| 15 | 20.74 ± 0.193 | 22.06 ± 0.153 | 20.41 ± 0.224 | 21.94 ± 0.124 | 17.80 ± 0.314 | 21.19 ± 0.170 | 17.94 ± 0.291 | 21.18 ± 0.154 |

a)

| **Generation** | **C0** | **C1** | **C2** | **ΔC0** | **ΔC1** | **ΔC2** |
| --- | --- | --- | --- | --- | --- | --- |
| 1 | 13.11 ± 0.031 | 13.15 ± 0.038 | 13.21 ± 0.055 | 13.49 ± 0.036 | 13.29 ± 0.036 | 13.80 ± 0.064 |
| 2 | 14.11 ± 0.040 | 14.22 ± 0.040 | 14.14 ± 0.046 | 14.61 ± 0.039 | 14.36 ± 0.046 | 14.91 ± 0.052 |
| 3 | 15.33 ± 0.044 | 15.35 ± 0.040 | 15.21 ± 0.068 | 15.56 ± 0.038 | 15.54 ± 0.041 | 15.88 ± 0.058 |
| 4 | 16.18 ± 0.042 | 16.33 ± 0.054 | 16.23 ± 0.060 | 16.54 ± 0.056 | 16.47 ± 0.055 | 16.74 ± 0.080 |
| 5 | 17.18 ± 0.052 | 17.34 ± 0.047 | 17.23 ± 0.063 | 17.39 ± 0.071 | 17.27 ± 0.062 | 17.87 ± 0.065 |
| 6 | 18.02 ± 0.071 | 18.21 ± 0.052 | 18.22 ± 0.065 | 18.35 ± 0.065 | 18.29 ± 0.060 | 18.54 ± 0.082 |
| 7 | 18.95 ± 0.078 | 18.97 ± 0.059 | 19.17 ± 0.074 | 19.16 ± 0.069 | 19.16 ± 0.060 | 19.50 ± 0.067 |
| 8 | 19.77 ± 0.076 | 19.68 ± 0.078 | 19.80 ± 0.084 | 19.85 ± 0.084 | 19.93 ± 0.055 | 20.23 ± 0.089 |
| 9 | 20.52 ± 0.080 | 20.57 ± 0.084 | 20.40 ± 0.084 | 20.57 ± 0.081 | 20.69 ± 0.069 | 20.78 ± 0.088 |
| 10 | 21.19 ± 0.087 | 21.10 ± 0.101 | 21.27 ± 0.092 | 21.25 ± 0.075 | 21.37 ± 0.079 | 21.71 ± 0.085 |
| 11 | 21.78 ± 0.095 | 21.97 ± 0.073 | 22.02 ± 0.089 | 21.85 ± 0.079 | 21.93 ± 0.088 | 22.30 ± 0.083 |
| 12 | 22.40 ± 0.105 | 22.53 ± 0.075 | 22.60 ± 0.107 | 22.61 ± 0.092 | 22.62 ± 0.088 | 22.90 ± 0.117 |
| 13 | 23.13 ± 0.101 | 23.28 ± 0.087 | 23.17 ± 0.095 | 23.19 ± 0.101 | 23.20 ± 0.095 | 23.49 ± 0.108 |
| 14 | 23.67 ± 0.102 | 23.77 ± 0.089 | 23.77 ± 0.122 | 23.84 ± 0.092 | 23.76 ± 0.098 | 24.13 ± 0.097 |
| 15 | 24.31 ± 0.096 | 24.25 ± 0.115 | 24.52 ± 0.107 | 24.37 ± 0.071 | 24.37 ± 0.094 | 24.75 ± 0.100 |

b)

| **Generation** | **M0 1-99** | **M0 5-95** | **M0 50-50** | **M0 95-5** | **M2 1-99** | **M2 5-95** | **M2 50-50** | **M2 95-5** |
| --- | --- | --- | --- | --- | --- | --- | --- | --- |
| 1 | 13.60 ± 0.026 | 13.95 ± 0.019 | 14.21 ± 0.015 | 14.24 ± 0.013 | 13.35 ± 0.035 | 13.58 ± 0.027 | 13.88 ± 0.026 | 13.60 ± 0.058 |
| 2 | 14.71 ± 0.025 | 14.99 ± 0.020 | 15.15 ± 0.018 | 15.05 ± 0.025 | 14.50 ± 0.039 | 14.79 ± 0.018 | 14.97 ± 0.017 | 14.52 ± 0.049 |
| 3 | 15.79 ± 0.021 | 16.04 ± 0.021 | 16.16 ± 0.018 | 16.03 ± 0.028 | 15.56 ± 0.031 | 15.91 ± 0.025 | 16.05 ± 0.017 | 15.48 ± 0.093 |
| 4 | 16.84 ± 0.028 | 17.04 ± 0.020 | 17.18 ± 0.018 | 16.88 ± 0.035 | 16.62 ± 0.035 | 16.90 ± 0.024 | 17.11 ± 0.019 | 15.35 ± 0.108 |
| 5 | 17.87 ± 0.022 | 18.08 ± 0.017 | 18.17 ± 0.020 | 17.75 ± 0.040 | 17.70 ± 0.036 | 17.94 ± 0.025 | 17.93 ± 0.021 | 15.83 ± 0.113 |
| 6 | 18.90 ± 0.029 | 19.12 ± 0.019 | 19.19 ± 0.022 | 18.62 ± 0.042 | 18.68 ± 0.041 | 18.97 ± 0.032 | 18.93 ± 0.023 | 16.25 ± 0.111 |
| 7 | 19.92 ± 0.028 | 20.13 ± 0.018 | 20.19 ± 0.021 | 19.46 ± 0.052 | 19.70 ± 0.041 | 19.96 ± 0.026 | 19.94 ± 0.024 | 16.54 ± 0.149 |
| 8 | 20.88 ± 0.031 | 21.10 ± 0.023 | 21.17 ± 0.026 | 20.21 ± 0.058 | 20.68 ± 0.043 | 20.94 ± 0.032 | 20.87 ± 0.027 | 16.81 ± 0.149 |
| 9 | 21.81 ± 0.030 | 22.01 ± 0.028 | 22.11 ± 0.026 | 20.98 ± 0.069 | 21.59 ± 0.050 | 21.86 ± 0.032 | 21.77 ± 0.035 | 17.16 ± 0.174 |
| 10 | 22.72 ± 0.036 | 22.95 ± 0.029 | 23.02 ± 0.028 | 21.72 ± 0.072 | 22.42 ± 0.065 | 22.80 ± 0.032 | 22.65 ± 0.038 | 17.44 ± 0.181 |
| 11 | 23.57 ± 0.037 | 23.82 ± 0.029 | 23.91 ± 0.031 | 22.38 ± 0.079 | 23.29 ± 0.064 | 23.66 ± 0.040 | 23.50 ± 0.036 | 17.54 ± 0.188 |
| 12 | 24.47 ± 0.038 | 24.70 ± 0.032 | 24.75 ± 0.037 | 23.02 ± 0.080 | 24.15 ± 0.063 | 24.54 ± 0.038 | 24.32 ± 0.039 | 17.84 ± 0.173 |
| 13 | 25.25 ± 0.046 | 25.54 ± 0.035 | 25.59 ± 0.038 | 23.64 ± 0.084 | 25.03 ± 0.062 | 25.36 ± 0.035 | 25.11 ± 0.046 | 18.15 ± 0.169 |
| 14 | 26.07 ± 0.041 | 26.33 ± 0.036 | 26.40 ± 0.041 | 24.30 ± 0.085 | 25.87 ± 0.063 | 26.21 ± 0.043 | 25.89 ± 0.047 | 18.37 ± 0.172 |
| 15 | 26.85 ± 0.056 | 27.14 ± 0.041 | 27.21 ± 0.044 | 24.83 ± 0.090 | 26.59 ± 0.063 | 26.97 ± 0.045 | 26.65 ± 0.049 | 18.64 ± 0.164 |

c)
