# Supplementary material for: Optimizing mating strategies to maximize genetic diversity in the mhorr gazelle (Nanger dama mhorr) ex situ breeding program
Source: BMC Zool. 2026 Apr 27;11:15. doi: 10.1186/s40850-026-00264-4 (PMC13154453; doi:10.1186/s40850-026-00264-4)
Supplement: Supplementary file 2 — Supplementary Material 2 [file 40850_2026_264_MOESM2_ESM.docx]

**SUPPLEMENTARY MATERIAL 2**

*Biodiversity and Conservation*

**Optimizing mating strategies to maximize genetic diversity in the mhorr gazelle (*Nanger dama mhorr*) ex situ breeding program**

Sonia Domínguez ^1^, Juan Pablo Gutiérrez ^2^, Eulalia Moreno ^1^ and Isabel Cervantes ^2^

^1^ Estación Experimental de Zonas Áridas-CSIC, Ctra. De Sacramento s/n, 04120 La Cañada de San Urbano, Almería, Spain

^2^ Department of Animal Production, Faculty of Veterinary, UCM, Avda. Puerta de Hierro s/n, 28040 Madrid, Spain

E-mail: [sdominguez@eeza.cisc.es](mailto:sdominguez@eeza.cisc.es)

**Table S2.** Evolution of inbreeding coefficient and its standard error throughout 15 generations of each mating strategy in the reference population of Almeria: strategies that minimize the coancestry between the parents (a), strategies that minimize the coancestry of the offspring (b) and mixed strategies (c).

| **Generation** | **F0** | **F1** | **ΔF0** | **ΔF1** | **Fw0** | **Fw1** | **ΔFw0** | **ΔFw1** |
| --- | --- | --- | --- | --- | --- | --- | --- | --- |
| 1 | 0.285 ± 0.0002 | 0.285 ± 0.0001 | 0.289 ± 0.0001 | 0.288 ± 0.0001 | 0.286 ± 0.0002 | 0.286 ± 0.0000 | 0.290 ± 0.0001 | 0.289 ± 0.0000 |
| 2 | 0.293 ± 0.0003 | 0.294 ± 0.0002 | 0.298 ± 0.0003 | 0.297 ± 0.0002 | 0.294 ± 0.0003 | 0.294 ± 0.0002 | 0.299 ± 0.0004 | 0.298 ± 0.0003 |
| 3 | 0.299 ± 0.0003 | 0.299 ± 0.0002 | 0.309 ± 0.0007 | 0.308 ± 0.0004 | 0.302 ± 0.0006 | 0.301 ± 0.0003 | 0.311 ± 0.0009 | 0.307 ± 0.0004 |
| 4 | 0.307 ± 0.0006 | 0.308 ± 0.0003 | 0.319 ± 0.0007 | 0.317 ± 0.0004 | 0.315 ± 0.0011 | 0.309 ± 0.0004 | 0.324 ± 0.0011 | 0.316 ± 0.0006 |
| 5 | 0.316 ± 0.0017 | 0.316 ± 0.0006 | 0.329 ± 0.0009 | 0.324 ± 0.0007 | 0.326 ± 0.0012 | 0.317 ± 0.0006 | 0.337 ± 0.0015 | 0.324 ± 0.0007 |
| 6 | 0.325 ± 0.0010 | 0.323 ± 0.0007 | 0.340 ± 0.0013 | 0.333 ± 0.0010 | 0.338 ± 0.0014 | 0.326 ± 0.0009 | 0.349 ± 0.0019 | 0.333 ± 0.0009 |
| 7 | 0.336 ± 0.0016 | 0.332 ± 0.0009 | 0.352 ± 0.0017 | 0.342 ± 0.0012 | 0.354 ± 0.0021 | 0.336 ± 0.0013 | 0.365 ± 0.0023 | 0.343 ± 0.0011 |
| 8 | 0.348 ± 0.0021 | 0.341 ± 0.0009 | 0.365 ± 0.0020 | 0.351 ± 0.0013 | 0.372 ± 0.0024 | 0.347 ± 0.0015 | 0.382 ± 0.0028 | 0.354 ± 0.0013 |
| 9 | 0.361 ± 0.0021 | 0.350 ± 0.0010 | 0.376 ± 0.0023 | 0.361 ± 0.0014 | 0.390 ± 0.0038 | 0.358 ± 0.0015 | 0.398 ± 0.0033 | 0.367 ± 0.0018 |
| 10 | 0.371 ± 0.0022 | 0.361 ± 0.0011 | 0.388 ± 0.0023 | 0.371 ± 0.0015 | 0.405 ± 0.0038 | 0.370 ± 0.0016 | 0.414 ± 0.0037 | 0.378 ± 0.0018 |
| 11 | 0.383 ± 0.0024 | 0.372 ± 0.0016 | 0.401 ± 0.0028 | 0.382 ± 0.0016 | 0.422 ± 0.0043 | 0.382 ± 0.0019 | 0.430 ± 0.0039 | 0.390 ± 0.0020 |
| 12 | 0.395 ± 0.0027 | 0.382 ± 0.0017 | 0.413 ± 0.0030 | 0.392 ± 0.0016 | 0.437 ± 0.0047 | 0.393 ± 0.0022 | 0.444 ± 0.0039 | 0.402 ± 0.0022 |
| 13 | 0.408 ± 0.0030 | 0.393 ± 0.0020 | 0.425 ± 0.0033 | 0.402 ± 0.0018 | 0.454 ± 0.0054 | 0.405 ± 0.0025 | 0.461 ± 0.0045 | 0.413 ± 0.0021 |
| 14 | 0.420 ± 0.0030 | 0.404 ± 0.0022 | 0.436 ± 0.0035 | 0.413 ± 0.0017 | 0.470 ± 0.0061 | 0.416 ± 0.0026 | 0.477 ± 0.0048 | 0.423 ± 0.0023 |
| 15 | 0.433 ± 0.0030 | 0.414 ± 0.0022 | 0.449 ± 0.0037 | 0.424 ± 0.0019 | 0.489 ± 0.0064 | 0.427 ± 0.0027 | 0.494 ± 0.0061 | 0.434 ± 0.0024 |

a)

| **Generation** | **C0** | **C1** | **C2** | **ΔC0** | **ΔC1** | **ΔC2** |
| --- | --- | --- | --- | --- | --- | --- |
| 1 | 0.306 ± 0.0006 | 0.306 ± 0.0007 | 0.297 ± 0.0009 | 0.306 ± 0.0006 | 0.307 ± 0.0006 | 0.308 ± 0.0011 |
| 2 | 0.312 ± 0.0007 | 0.311 ± 0.0007 | 0.306 ± 0.0008 | 0.312 ± 0.0007 | 0.315 ± 0.0007 | 0.312 ± 0.0009 |
| 3 | 0.314 ± 0.0007 | 0.314 ± 0.0006 | 0.311 ± 0.0011 | 0.319 ± 0.0007 | 0.318 ± 0.0007 | 0.318 ± 0.0009 |
| 4 | 0.321 ± 0.0006 | 0.320 ± 0.0008 | 0.316 ± 0.0009 | 0.324 ± 0.0009 | 0.324 ± 0.0009 | 0.325 ± 0.0012 |
| 5 | 0.326 ± 0.0007 | 0.324 ± 0.0007 | 0.321 ± 0.0009 | 0.331 ± 0.0010 | 0.331 ± 0.0009 | 0.327 ± 0.0009 |
| 6 | 0.332 ± 0.0010 | 0.330 ± 0.0007 | 0.325 ± 0.0009 | 0.335 ± 0.0009 | 0.335 ± 0.0008 | 0.335 ± 0.0011 |
| 7 | 0.336 ± 0.0011 | 0.337 ± 0.0008 | 0.330 ± 0.0009 | 0.341 ± 0.0009 | 0.340 ± 0.0008 | 0.339 ± 0.0009 |
| 8 | 0.342 ± 0.0010 | 0.343 ± 0.0010 | 0.338 ± 0.0011 | 0.348 ± 0.0011 | 0.346 ± 0.0007 | 0.346 ± 0.0011 |
| 9 | 0.348 ± 0.0010 | 0.348 ± 0.0011 | 0.346 ± 0.0010 | 0.354 ± 0.0010 | 0.351 ± 0.0009 | 0.354 ± 0.0011 |
| 10 | 0.354 ± 0.0011 | 0.356 ± 0.0013 | 0.350 ± 0.0011 | 0.360 ± 0.0009 | 0.358 ± 0.0009 | 0.357 ± 0.0010 |
| 11 | 0.361 ± 0.0011 | 0.359 ± 0.0009 | 0.356 ± 0.0010 | 0.367 ± 0.0010 | 0.365 ± 0.0011 | 0.363 ± 0.0010 |
| 12 | 0.368 ± 0.0012 | 0.366 ± 0.0009 | 0.362 ± 0.0013 | 0.371 ± 0.0011 | 0.370 ± 0.0010 | 0.370 ± 0.0015 |
| 13 | 0.372 ± 0.0011 | 0.371 ± 0.0011 | 0.369 ± 0.0011 | 0.377 ± 0.0012 | 0.376 ± 0.0011 | 0.376 ± 0.0012 |
| 14 | 0.379 ± 0.0012 | 0.378 ± 0.0010 | 0.375 ± 0.0014 | 0.382 ± 0.0011 | 0.382 ± 0.0011 | 0.381 ± 0.0011 |
| 15 | 0.384 ± 0.0011 | 0.385 ± 0.0013 | 0.379 ± 0.0012 | 0.388 ± 0.0009 | 0.388 ± 0.0010 | 0.386 ± 0.0011 |

b)

| **Generation** | **M0 1-99** | **M0 5-95** | **M0 50-50** | **M0 95-5** | **M2 1-99** | **M2 5-95** | **M2 50-50** | **M2 95-5** |
| --- | --- | --- | --- | --- | --- | --- | --- | --- |
| 1 | 0.297 ± 0.0004 | 0.291 ± 0.0002 | 0.286 ± 0.0002 | 0.285 ± 0.0002 | 0.295 ± 0.0006 | 0.290 ± 0.0004 | 0.281 ± 0.0002 | 0.276 ± 0.0005 |
| 2 | 0.301 ± 0.0004 | 0.297 ± 0.0002 | 0.293 ± 0.0002 | 0.292 ± 0.0002 | 0.300 ± 0.0006 | 0.295 ± 0.0002 | 0.289 ± 0.0002 | 0.287 ± 0.0002 |
| 3 | 0.307 ± 0.0003 | 0.303 ± 0.0002 | 0.300 ± 0.0002 | 0.299 ± 0.0003 | 0.306 ± 0.0005 | 0.300 ± 0.0003 | 0.295 ± 0.0002 | 0.297 ± 0.0014 |
| 4 | 0.311 ± 0.0003 | 0.308 ± 0.0003 | 0.305 ± 0.0002 | 0.307 ± 0.0004 | 0.310 ± 0.0005 | 0.306 ± 0.0003 | 0.301 ± 0.0002 | 0.322 ± 0.0017 |
| 5 | 0.316 ± 0.0003 | 0.313 ± 0.0002 | 0.311 ± 0.0002 | 0.314 ± 0.0004 | 0.314 ± 0.0005 | 0.311 ± 0.0003 | 0.309 ± 0.0003 | 0.336 ± 0.0019 |
| 6 | 0.320 ± 0.0004 | 0.317 ± 0.0002 | 0.315 ± 0.0002 | 0.321 ± 0.0005 | 0.319 ± 0.0005 | 0.315 ± 0.0004 | 0.314 ± 0.0003 | 0.349 ± 0.0020 |
| 7 | 0.324 ± 0.0003 | 0.321 ± 0.0002 | 0.319 ± 0.0003 | 0.327 ± 0.0006 | 0.323 ± 0.0005 | 0.320 ± 0.0003 | 0.318 ± 0.0003 | 0.365 ± 0.0027 |
| 8 | 0.328 ± 0.0004 | 0.325 ± 0.0003 | 0.323 ± 0.0003 | 0.334 ± 0.0007 | 0.327 ± 0.005 | 0.324 ± 0.0004 | 0.323 ± 0.0003 | 0.379 ± 0.0028 |
| 9 | 0.332 ± 0.0004 | 0.329 ± 0.0003 | 0.327 ± 0.0003 | 0.340 ± 0.0008 | 0.331 ± 0.0006 | 0.328 ± 0.0004 | 0.327 ± 0.0004 | 0.391 ± 0.0032 |
| 10 | 0.336 ± 0.0004 | 0.333 ± 0.0003 | 0.331 ± 0.0003 | 0.346 ± 0.0009 | 0.336 ± 0.0008 | 0.332 ± 0.0004 | 0.332 ± 0.0004 | 0.404 ± 0.0034 |
| 11 | 0.340 ± 0.0005 | 0.337 ± 0.0003 | 0.335 ± 0.0004 | 0.352 ± 0.0010 | 0.340 ± 0.0007 | 0.336 ± 0.0004 | 0.336 ± 0.0004 | 0.420 ± 0.0036 |
| 12 | 0.343 ± 0.0005 | 0.341 ± 0.0003 | 0.340 ± 0.0004 | 0.359 ± 0.0010 | 0.344 ± 0.0007 | 0.340 ± 0.0004 | 0.341 ± 0.0004 | 0.430 ±0.0033 |
| 13 | 0.348 ± 0.0005 | 0.345 ± 0.0004 | 0.344 ± 0.0004 | 0.365 ± 0.0010 | 0.348 ± 0.0006 | 0.344 ± 0.0004 | 0.345 ± 0.0005 | 0.441 ± 0.0032 |
| 14 | 0.352 ± 0.0005 | 0.349 ± 0.0004 | 0.348 ± 0.0004 | 0.370 ± 0.0010 | 0.351 ± 0.0007 | 0.348 ± 0.0005 | 0.350 ± 0.0005 | 0.452 ± 0.0032 |
| 15 | 0.356 ± 0.0006 | 0.353 ± 0.0004 | 0.351 ± 0.0005 | 0.377 ± 0.0011 | 0.356 ± 0.0007 | 0.352 ± 0.0005 | 0.354 ± 0.0015 | 0.462 ± 0.0031 |

c)
