# Supplementary material for: Optimizing mating strategies to maximize genetic diversity in the mhorr gazelle (Nanger dama mhorr) ex situ breeding program
Source: BMC Zool. 2026 Apr 27;11:15. doi: 10.1186/s40850-026-00264-4 (PMC13154453; doi:10.1186/s40850-026-00264-4)
Supplement: Supplementary file 3 — Supplementary Material 3 [file 40850_2026_264_MOESM3_ESM.docx]

**SUPPLEMENTARY MATERIAL 3**

*BMC Zoology*

**Optimizing mating strategies to maximize genetic diversity in the mhorr gazelle (*Nanger dama mhorr*) ex situ breeding program**

Sonia Domínguez ^1^, Juan Pablo Gutiérrez ^2^, Eulalia Moreno ^1^ and Isabel Cervantes ^2^

^1^ Estación Experimental de Zonas Áridas-CSIC, Ctra. De Sacramento s/n, 04120 La Cañada de San Urbano, Almería, Spain

^2^ Department of Animal Production, Faculty of Veterinary, UCM, Avda. Puerta de Hierro s/n, 28040 Madrid, Spain

E-mail: [sdominguez@eeza.cisc.es](mailto:sdominguez@eeza.cisc.es)

**Table S3.** Confidence intervals of generations 1, 5 and 15 for each mating strategy in the reference population of Almeria. Confidence intervals were obtained by multiplying the standard error of the effective population size by 1.96.

| **Strategy** | **Generation 1** | **Generation 5** | **Generation 15** |
| --- | --- | --- | --- |
| **F0** | 14.1892 – 14.2462 | 17.4840 – 17.6857 | 20.3661 – 21.1236 |
| **F1** | 14.1989 – 14.2531 | 17.6171 – 17.8024 | 21.7616 – 22.3613 |
| **ΔF0** | 14.6661 – 14.7335 | 17.6515 – 17.8772 | 19.9680 – 20.8450 |
| **ΔF1** | 14.5416 – 14.5960 | 17.8792 – 18.0342 | 21.6999 – 22.1875 |
| **Fw0** | 14.1265 – 14.1666 | 16.9493 – 17.2724 | 17.1852 – 18.4166 |
| **Fw1** | 14.1142 – 14.1505 | 17.5894 – 17.7617 | 20.8597 – 21.5270 |
| **ΔFw0** | 14.6044 – 14.7061 | 17.0217 – 17.3697 | 17.3706 – 18.5099 |
| **ΔFw1** | 14.4909 – 14.5452 | 17.7411 – 17.9209 | 20.8816 – 21.4862 |
| **C0** | 14.0524 – 13.1733 | 17.1216 – 17.2425 | 24.1189 – 24.4968 |
| **C1** | 13.0802 – 13.2279 | 17.2471 – 17.4330 | 24.0246 – 24.4758 |
| **C2** | 13.0970 – 13.3141 | 17.1064 – 17.3543 | 24.3086 – 24.7293 |
| **ΔC0** | 13.4236 – 13.5656 | 17.2540 – 17.5308 | 24.2272 – 24.5049 |
| **ΔC1** | 13.2209 – 13.3636 | 17.1473 – 17.3901 | 24.1821 – 24.5492 |
| **ΔC2** | 13.6699 – 13.9202 | 17.7412 – 17.9970 | 24.5494 – 24.9424 |
| **M0 1-99** | 13.5505 – 13.6526 | 17.8300 – 17.9162 | 26.7442 – 26.9620 |
| **M0 5-95** | 13.9103 – 13.9834 | 18.0488 – 18.1160 | 27.0624 – 27.2214 |
| **M0 50-50** | 14.1808 – 14.2409 | 18.1297 – 18.2074 | 27.1207 – 27.2935 |
| **M0 95-5** | 14.2116 – 14.2645 | 17.6719 – 17.8276 | 24.6499 – 25.0021 |
| **M2 1-99** | 13.2767 – 13.4145 | 17.6317 – 17.7744 | 26.4634 – 26.7100 |
| **M2 5-95** | 13.5293 – 13.6341 | 17.8919 – 17.9884 | 26.8779 – 26.7100 |
| **M2 50-50** | 13.8309 – 13.9347 | 17.8934 – 17.9740 | 26.5520 – 26.7455 |
| **M2 95-5** | 13.4817 – 13.7091 | 15.6050 – 16.0482 | 18.3144 – 18.9573 |
